# Overuse injuries in sport: a comprehensive overview

**DOI:** 10.1186/s13018-018-1017-5

**Published:** 2018-12-05

**Authors:** R. Aicale, D. Tarantino, N. Maffulli

**Affiliations:** 1 Department of Musculoskeletal Disorders, School of Medicine and Dentistry, University of Salerno, Salerno, Italy; 2 Barts and the London School of Medicine and Dentistry, Centre for Sports and Exercise Medicine, Mile End Hospital, Queen Mary University of London, 275 Bancroft Road, London, E1 4DG England

**Keywords:** Fractures, Stress fracture, Overuse injuries, Osteochondritis dissecans, Tendinopathy

## Abstract

**Background:**

The absence of a single, identifiable traumatic cause has been traditionally used as a definition for a causative factor of overuse injury. Excessive loading, insufficient recovery, and underpreparedness can increase injury risk by exposing athletes to relatively large changes in load. The musculoskeletal system, if subjected to excessive stress, can suffer from various types of overuse injuries which may affect the bone, muscles, tendons, and ligaments.

**Methods:**

We performed a search (up to March 2018) in the PubMed and Scopus electronic databases to identify the available scientific articles about the pathophysiology and the incidence of overuse sport injuries. For the purposes of our review, we used several combinations of the following keywords: overuse, injury, tendon, tendinopathy, stress fracture, stress reaction, and juvenile osteochondritis dissecans.

**Results:**

Overuse tendinopathy induces in the tendon pain and swelling with associated decreased tolerance to exercise and various types of tendon degeneration. Poor training technique and a variety of risk factors may predispose athletes to stress reactions that may be interpreted as possible precursors of stress fractures. A frequent cause of pain in adolescents is juvenile osteochondritis dissecans (JOCD), which is characterized by delamination and localized necrosis of the subchondral bone, with or without the involvement of articular cartilage. The purpose of this compressive review is to give an overview of overuse injuries in sport by describing the theoretical foundations of these conditions that may predispose to the development of tendinopathy, stress fractures, stress reactions, and juvenile osteochondritis dissecans and the implication that these pathologies may have in their management.

**Conclusions:**

Further research is required to improve our knowledge on tendon and bone healing, enabling specific treatment strategies to be developed for the management of overuse injuries.

## Background

The specific definition of overuse injury was most commonly based on the concept of an injury occurring in the absence of a single, identifiable traumatic cause [1]. Professional soccer players sustain on average 2.0 injuries per season, which cause them to miss 37 days in a 300-day season on average [2]. Following the updated injury etiology model, training and match load contribute, together with intrinsic and extrinsic risk factors, to the multifactorial and dynamic etiology of injury [3]. Not only excessive loading and insufficient recovery, but also underpreparedness may increase injury risk by exposing players to relatively large changes, or spikes, in load during periods with higher training and match loads [4].

The tendons transfer the force produced from muscular contraction to the bone. In most instances, sports-related tendinopathies present well-defined histopathological lesions, providing an explanation for the chronicity of symptoms which often occur in athletes with tendinopathies [5–8].

The aim of the present article is to investigate the physiopathology, clinical presentation, diagnostic tools, and management of the most common overuse sport injuries. In particular, we focus on tendinopathy, stress reaction, stress fracture, and juvenile osteochondritis dissecans, which are the most frequent lesion caused by overuse. Furthermore, in the first part of this study, to better understand the changes of the bone, muscle, and tendon structures, we mention different mechanisms present in an overuse situation.

Overuse tendinopathy induces in the affected tendon pain and swelling, and associated decreased load tolerance and function during exercise of the limb [9, 10]. Various types of tendon degeneration have been described at electron microscopy, namely (a) hypoxic degeneration, (b) hyaline degeneration, (c) mucoid or myxoid degeneration, (d) fibrinoid degeneration, (e) lipoid degeneration, (f) calcification, and (g) fibrocartilaginous and bony metaplasia [11]. Healing of tendinopathic tendons relies on the intrinsic ability of tenocytes to respond to the stimulus induced by the injury to the surrounding tissue matrix [12, 13] and consists of a cellular response including apoptosis (programmed cell death), chemotaxis, proliferation, and differentiation [14]. The mechanism underlying the precise sequence of these events, which balance the effectiveness of healing and any subsequent predisposition to repetitive damage, remains obscure.

The essence of tendinopathy is a “failed healing response.” This model suggests that, after an acute insult to the tendon, an early inflammatory response that would normally result in successful injury resolution veers toward an ineffective healing response [15], with degeneration and proliferation of tenocytes, disruption of collagen fibers, and subsequent increase in non-collagenous matrix [7, 16–19] (Table 1).

**Table 1:** Types of tendon collagens

Types of collagens	MW	Quantity (%)	Localization
Type I	95.000	97–98	Endotenon, epitenon, paratenon, MJT
Type II	95.000	0.2–0.8	OTJ cartilage
Type III	95.000	1–1.5	Endotenon, paratenon, vessel walls, MTJ
Type IV	180.000	< 0.2	Basal membrane of vessels, MTJ
Type V	300.000	< 0.2	Vessel walls, MTJ

*MW* molecular weight, *MTJ* muscletendon junction, *OTJ* osteotendon junction

Poor training technique and a variety of risk factors may predispose players to lower limb overuse injuries affecting the bone, including stress reactions to full-fledged stress fractures. The underlying principle of the bone response to stress is Wolff’s law, whereby changes in the stresses imposed on the bone lead to changes in its internal architecture [20, 21]. Stress fractures, defined as microfractures of the cortical bone tissue, affect thousands of athletes per year [22, 23]. Certain subpopulations, including runners, gymnasts, and female athletes, exhibit higher rates of stress fractures [24, 25]. If left untreated, a stress fracture can progress to a complete fracture of a bone, which may require surgical fixation [26]. In addition, factors contributing to stress fractures increase the risk for osteoporosis, a substantial long-term health concern [27].

Stress reactions of the musculoskeletal system may be interpreted as possible precursors of stress fractures. Biological tissues, in contrast to artificial products, can react in numerous and complex ways. This can lead not only to a continual weakening of the tissue, but also to adaption phenomena in response to overuse. The causes of such stress reactions are still unclear.

Juvenile osteochondritis dissecans (JOCD) is a frequent cause of pain in adolescents, both athletes and non-athletes. JOCD is characterized by delamination and localized necrosis of the subchondral bone, with or without the involvement of the overlying articular cartilage [28–31]. The etiology remains unclear, but repetitive microtrauma, such as that typical of overuse injury, is considered the significant factor leading to JOCD [28, 29].

## The role of inflammation and molecular factors in overuse injuries

### The effects of an altered inflammatory response

In this model, the question arises as to why the healing response is successful in some individuals but fails in others. More importantly, can we identify factors which may increase the risk of this ineffective healing response? For example, the incidence of tendinopathy is increased in individuals with obesity and decreased insulin sensitivity, as seen in patients with type 1 and type 2 diabetes mellitus (T1/T2DM) [10, 32–34]. Evidence for a chronic, low-grade inflammatory state in obesity is represented principally by marked increases in plasma levels of proinflammatory cytokines such as tumor necrosis factor (TNF)-a and interleukin (IL)-6, and proinflammatory chemokines such as monocyte chemoattractant protein (MCP)-1 [34].

Patients with type 1 and type 2 diabetes exhibit a less effective healing response [35]. Recently, it has been demonstrated the presence of an independent relationship between impaired insulin sensitivity and the development of chronic low-grade inflammation through a protein, the levels of which are normally physiologically inhibited by insulin, called FOXO1, a key upregulator of the proinflammatory cytokine IL-b [35].

Considering the influence that a prolonged state of low-grade systemic inflammation may have on the healing process after acute tendon injury, it must be appreciated that tendon healing is a delicate and prolonged process even under optimal physiological conditions [10, 34]. Even minor disruptions to any of the noted healing stages could result in a much more prolonged and complicated resolution of injury. Similarly, if several minor disruptions to this process occur (in the form, for example, of microtraumas), complete healing and resolution of injury become progressively unlikely [10].

The acute inflammatory phase noted in the first few days after a tendon injury is marked by the migration of inflammatory cells such as macrophages and monocytes [36]. As the chronic inflammatory state in obesity is associated with a reduction in the numbers of circulating macrophages [33], such a decrease in the availability of circulating cells may result in the mounting of a less effective early healing response.

Such findings are consistent with a post-injury state of “failed healing,” in which evidence of matrix disorganization, increased amounts of extracellular ground substance, and a degree of separation between collagen fibers has been noted [37, 38], with associated greater vulnerability to future mechanical strain [10].

This relationship may help to explain the influence that mechanical overuse plays in the development of tendinopathy. Examining the incidence of tendinopathy among patients with type 2 diabetes, unilateral or bilateral tendinopathy was found in 32% of the diabetic patients studied versus 10% of controls [39]. Also, when the incidence of unilateral tendinopathy among diabetic patients was examined more closely, 45% were found to occur in the right shoulder compared with just 27% in the left shoulder [33].

### Molecular factors in overuse injury

A lack of exposure to adequate levels of physiological stress over a prolonged time period or “underloading” may paradoxically predispose to overload injury [34]. An underloaded tendon may become unable to cope with increased demands imposed on it. Thus, underuse of a tendon may result in an imbalance between matrix metalloproteinases and their inhibitors (tissue inhibitors of matrix metalloproteinases), with resultant tendon degradation [34].

Molecular agents may link the events of tendon degeneration and ineffective tendon healing with the production and persistence of reactive oxygen species within both the intra- and extra-cellular milieu of the tendon tissue [40]. The reactive oxygen production is strongly influenced by lifestyle factors, e.g., nutrition and the intensity and frequency of exercise.

The term reactive oxygen species (ROS) encompasses reactive species derived from oxygen. A free radical is any species capable of independent existence that contains one or more unpaired electrons [41]. Physiologically relevant ROS include the superoxide anion (O^2−^), hydrogen peroxide (H_2_O_2_), hydroxyl radical (HO^−^), singlet oxygen (^1^O_2_), and peroxyl radicals (RO^2−^). An inter-related group of radical and non-radical reactive species is the reactive nitrogen species (RNS) [42–44].

The principal site for ROS formation in non-stressed cells is the mitochondrial respiratory chain. This series of coupled redox reactions leads to the formation of ATP with molecular oxygen the ultimate electron acceptor and being reduced to water [45, 46].

ROS may mediate processes of cell proliferation [47], differentiation [48], and adaptive responses [45, 49]. At higher levels, ROS may initiate and/or execute the demise of the cell through programmed as well as necrotic cell death mechanisms [50–52].

Traditionally, ROS are viewed as imposing cellular and tissue damage via lipid peroxidation, DNA damage, and protein modification [45, 53]. However, in themselves, O^2−^ and H_2_O_2_ are not potent biological oxidizers [45], although certain proteins may be prone to direct modification by these species. ROS production is involved in various cancers (e.g., the lung, colon), coronary heart disease, autoimmune diseases, etc. [41]. Furthermore, ROS are implicated in overuse exercise-related damage in muscle [49] and may impair fracture healing in the bone [54].

Cells and tissues contain many antioxidant molecules, but many antioxidants are also capable of acting as pro-oxidants [55]. For example, ascorbic acid in the presence of iron/copper generates HO^−^, and with flavonoids may generate O^2−^ [55].

ROS may be involved in tendinopathies or other stress reactions: indeed, synthesis, structure, and integrity of connective tissues are influenced by them. Reactive species may be produced within the intra- and extra-tendinous environment. Evidence suggest that ROS constitute a stress factor during not-hard exercise [49]. Excessive exercise induces elevated ROS production, primarily from mitochondria [56]. Exercise also stimulates the immune response [56–58], with increased leucocyte numbers, in particular granulocytes. Exhaustive exercise in cross-country skiers produced neutrophil mobilization and increased ROS generation on subsequent stimulation. Enhanced phagocytic O^2−^ generation accurs approximately 24 h after exhaustive exercise [59].

Increased phagocyte activity probably does not contribute to elevated ROS production during short-term exercise, but may act as a secondary source of ROS during recovery from heavy exercise [59].

During cyclical tendon loading, the period of maximum tensile load is associated with ischemia, and relaxation with reperfusion. This restoration of normal tissue oxygenation may lead to enhanced ROS production [59, 60]. There is potential for re-oxygenation resulting in a cycle of enhanced ROS production, most probably at sublethal levels within the non-degenerate tendon [40].

Hyperthermia is a feature of tendon use inducing ROS production. During exercise, the central core temperature of the muscles can exceed 47 °C [49], a temperature resulting in increased ROS production in mitochondria [49]. Similarly, in exercising the tendon, core temperatures may reach 45 °C, contributing to their damage [60].

During fibrogenesis, ROS, primarily derived from specialized phagocytes and products arising from lipid peroxidation, induce overexpression of fibrogenic cytokines and increase the synthesis of collagen [61]. Endogenous and exogenous ROS may also exert effects on tenocyte proliferation, development, and viability, with implications on both tendinopathy and post-rupture healing [62]. Tenocytes are motile and highly proliferative and rapidly increase in number following injury [62].

Tenocyte numbers are altered in degenerated tendons, and the selective deletion of tenocytes from damaged tendon may be a factor in degeneration, but also a prerequisite to healing. Heightened levels of ROS production may not only induce cell death, but also determine the mechanistic form of that death, in particular, the ratio of programed cell death (PCD): necrosis [51].

High concentrations of H_2_O_2_ can prevent apoptosis. Conversely, “bursts” of ROS [51, 63] and reductions in antioxidant enzyme activity [64] frequently accompany the induction of apoptosis, and oxidative stress is a common feature of the late phase of apoptosis [51]. For example, the pro-apoptotic transcription factor p53 demonstrates impaired DNA binding following exposure to ROS. However, it can induce apoptosis by induction of enhanced mitochondrial ROS generation [65].

## Overuse sport injuries

### Tendinopathy: consideration and management

Tendinopathy has been hypothesized to result from inflammatory changes in the tendon, and secondary to its frequent or excessive use, assigning the label of “tendinitis” or “tendonitis” to such a presentation [9, 10, 66]. However, anti-inflammatory agents are largely unsuccessful in the treatment of the condition [15, 66], and with the increase in histopathological data showing degenerative changes but little inflammation, the inflammatory hypothesis in overuse tendon injury became decreasingly popular [10, 15, 36, 67]. The term “tendonitis” became increasingly replaced by “tendinosis” [36], but a definitive diagnosis of either should only be made following histopathological confirmation [15, 36, 67].

However, it became evident that tendon biopsies from operated patients were likely to represent the end stage of a pathological continuum [10], probably demonstrating a different histopathological picture to that which would be seen in the initial stages of injury [36, 67]. This was supported by evidence from human and animal biopsies that showed that both peritendinitis and a failed healing response, wrongly labeled “tendinosis,” could be present concurrently [36].

In tendinopathic lesions, the parallel orientation of collagen fibers is lost, with a decrease in collagen fiber diameter and in the overall density of collagen. Collagen microtears may also occur and may be surrounded by erythrocytes, fibrin, and fibronectin deposits. Normally, collagen fibers in tendons are tightly bundled in a parallel fashion. In tendinopathic samples, there is unequal and irregular crimping, loosening, and increased waviness of collagen fibers, with an increase in type III (reparative) collagen [17, 19, 68–71]. Vascularity is typically increased, and blood vessels are randomly oriented, sometimes perpendicular to collagen fibers [72, 73]. Inflammatory lesions [72–75] and granulation tissue [74, 75] are infrequent and, when found, are associated with partial rupture: therefore, tenocytes are abnormally plentiful in some areas [76, 77].

Tendinopathies are common in elite and recreational athletes and are traditionally considered overuse injuries, involving excessive tensile loading and subsequent breakdown of the loaded tendon [78, 79]. Although acute traumatic conditions such as ligament and muscle tears receive much attention in the lay press, tendinopathies account for much of the lost time in practice and competition [80, 81].

Biopsy studies have shown that classic inflammatory changes are not frequently seen in chronic tendon conditions and that histopathology features in tendinopathic tendons are clearly different from normal tendons [82, 83].

All tendons can develop tendinopathy [5, 84]. The supraspinatus, common wrist extensor, quadriceps, patellar, posterior tibialis, and Achilles tendons are probably the most commonly affected tendons. Insertional tendinopathy is one of the most common forms of tendinopathy, and, in particular, the supraspinatus, common wrist extensor, quadriceps, and patellar tendons are most affected by it [84]. The Achilles tendon, on the other hand, can present tendinopathy of the main body of the tendon, paratendinopathy and insertional tendinopathy, each with different clinical features and management implications [84].

Achilles tendinopathy (AT) is a common overuse injury among athletes, with an increasing incidence over the past 30 years [85, 86]. AT is particularly prevalent in athletes whose sport involve running and jumping activities [87, 88] and is thus common in sports such as soccer. In the four principal soccer leagues in England, there are an average of 3.5 Achilles tendon-related injuries per week in the preseason and an average of one injury per week in the competitive season [89, 90].

Tendinopathies may result from excessive loading of the tendon and subsequent mechanical breakdown of the loaded tendon [91]. Theoretically, repeated microinjuries may occur, and the tendon may be able to heal a certain level of microinjury. However, as training and heavy loading of the tendon continues, this healing process may be overwhelmed, and a further injury ensues.

Other factors in addition to training errors may lead to increased loading of the tendon, such as poor technique [92, 93] or inadequate athletic equipment [94]. Also, intrinsic factors, such as the status of the muscles, ligaments, and bones surrounding the tendon, may alter the level of the load on the tendon [19]. Recent biomechanical studies about failure modes of the muscle-tendon units have shown that failure occurs within the muscle near the muscle-tendon junction [95, 96].

Relatively, little is known about the role of neuronal regulation in tendinopathy, and the source of pain has not been clarified yet [97]. The presence of pain in tendinopathy requires not only mechanical changes, but also alterations in the way the local cells and the peripheral nerves react to this change. A recent systematic review showed that the peripheral neuronal phenotype is altered in tendinopathy [97] and that the peripheral and central pain processing pathways are important factors in the pathogenesis of painful human tendinopathy. Changes in the peripheral neuronal phenotype may be the primary source of pain [97]^.^

Clinical history and examination are essential for diagnosis. Clinically, tendinopathy is characterized by pain, swelling (diffuse or localized), and impaired performance [6].

Pain is the cardinal symptom, and it occurs at the beginning and a short while after the end of a training session. As the pathological process progresses, pain may occur during the entire exercise session, and, in severe cases, it may interfere with the activities of daily living. Clinical examination is the best diagnostic tool. In tendinopathy of the main body of the Achilles tendon, the location of pain is 2–6 cm above the insertion into the calcaneum, and pain on palpation is a reliable and accurate test for diagnosis [98].

In addition to the swelling on the posteromedial aspect of the tendon and palpation pain, some clinical tests have been described for non-insertional AT diagnosis. They can be divided into palpation tests (tendon thickening, crepitus, pain on palpation, the Royal London Hospital (RLH) test, the painful arc sign) and tendon loading tests (pain on passive dorsiflexion, pain on single heel raise, and pain on hopping).

Plain radiography can be used to diagnose associated or incidental bony abnormalities [99].

Ultrasound is an effective imaging method since it correlates well with the histopathologic findings despite being an operator-dependent [100]. MRI studies should be performed only if the ultrasound scan remains unclear. The ultrasound (US) signs of hypoechoic areas, spindle-shaped thickening, neovascularization, and paratenon blurring [101] are associated with AT [102] and may be potential predictors of future tendinopathy [87] when present in asymptomatic individuals.

The first line of management for AT is conservative, and different treatments such as nonsteroidal anti-inflammatory drugs, physical therapy, taping, cryotherapy, shock wave therapy, hyperthermia, and various peritendinous injections have been used with varying success [103]. The management of AT lacks strong evidence-based support, because few treatment modalities have been investigated in randomized controlled trials [103], and approximately 25% of patients do not respond to conservative management [104]. Good results have been reported with eccentric exercises [105, 106], but these alone may not work in all patients [107], and their mechanism of action is not completely understood [106]. These are the most effective conservative treatment for non-insertional AT. The most commonly used protocol is the Alfredson’s protocol: the exercises are performed in three sets of 15 repetitions, twice a day for 12 weeks [108].

ESWT, when compared with eccentric strengthening in a RCT, showed comparable outcomes, with 60% of the patients at least significantly improved in both of the treatment groups, and significantly better than those in the “wait and see” control group [109]. Where available, ESWT should probably be a second-line treatment.

Various injection therapies have been proposed [110]. In a recent systematic review [111], only ultrasound-guided sclerosing polidocanol injections seemed to yield promising results, but these results do not appear to have been duplicated outside Scandinavia [112]: indeed, Ebbesen et al. [113], in a RTC, concludes that polidocanol injections are a safe treatment, but in the mid-term, the effects are the same of a placebo treatment for chronic Achilles tendinopathy. The use of platelet-rich plasma (PRP) is growing exponentially, especially among sports medicine physicians, but the only well-designed RCT published on PRP in AT showed no significant difference in pain or activity level between PRP and saline injection at 6, 12, or 24 weeks when combined with an eccentric stretching program [114]. High-volume image-guided injections (HVIGI) significantly reduce pain and improve function in patients with resistant AT [115]. A recent study found relevant clinical results with the contemporaneous administration of platelet-rich plasma and high-volume image-guided injections of saline treatments, which influence tendon repair by different mechanisms and grants a greater improvement for patellar tendinopathy [116].

Conservative treatment fails in between one quarter and one third of patients, and surgical intervention is required [117]. Minimally invasive therapies which strip the paratenon from the tendon, either directly [118] or indirectly with high-volume fluid injection [115], have shown good initial results in relieving the symptoms of non-insertional AT [103, 119].

Another technique consists in multiple percutaneous longitudinal tenotomies, which can be performed under ultrasound guidance [120, 121]. Minimally invasive surgical treatment would appear to be a useful intermediate step between failed conservative treatment and formal open surgery [103].

The high recurrence rate (27%) for AT when managed conservatively reflects the chronic and recurrent character of this condition. The frequent relapse of symptoms when players return to football after a short rehabilitation period could be explained if the pain is only the tip of the iceberg. Therefore, it could be suggested that a longer rehabilitation period at the first signs of AT could be beneficial to avoid recurrences [122].

### Stress reaction and stress fracture

Stress reactions may be interpreted as precursors of stress fractures [123]. The causes of such stress are still unclear. For example, it is unknown to what extent a predisposition to these stress symptoms by mechanical stress alone or whether other factors such as physical condition, nutrition, or even hormone balance come in to play. Early diagnosis considerably reduces the impairment of the healing process. The treatment of a stress reaction should be the same as for a diagnosed stress fracture [123]. Much of our epidemiological knowledge about stress fractures originates from research on military recruits [124] and high school athletes [25].

The response of the bone to repetitive stress is increased osteoclastic activity over osteoblastic new bone formation, which results in temporary weakening of the bone [125]. The eventual adaptive response is periosteal new bone formation to provide reinforcement [126]. However, if physical stress continues, an osteoclastic activity may predominate, resulting initially in microfractures (commonly seen as bone marrow edema on MRI, consistent with a stress reaction), and eventually, a true cortical break (stress fracture) may result [1]. If strain becomes excessive or adequate rest is not implemented, stress reaction and eventually a stress fracture can results [126, 127].

There is a difference between stress fractures from fatigue and insufficiency type. Fatigue fractures are the typical overuse stress fractures observed in athletes and military recruits with normal bone density. They result from an imbalance in the ability of the bone to keep up with skeletal repair from an excessive bone strain with progressive accumulation of microdamage [126]. An insufficiency fracture is seen in those with low bone mineral density (BMD), such as runners with the female athlete triad; metabolic bone disease; or osteoporosis. Insufficiency fractures result from poor bone remodeling (increased resorption and depressed formation) in response to normal strain [126].

Rizzone et al. [128] investigated the epidemiology of stress fractures in 671 collegiate student-athletes for the academic years 2004–2005 through 2013–2014. The rate of stress fracture was highest among endurance athletes and higher in women than in men. Higher rates among female athletes were found not only in cross-country athletes, indoor track, and outdoor track athletes, but also in basketball and soccer athletes. Twenty-two percent of stress fractures were recurrent, and 20% resulted in season-ending injuries [128].

The number of reports in the literature of lower extremity stress fractures in female soccer athletes is small [129]. Of the 18 million Americans who play soccer, 78% are younger than 18 years and more than 40% are female [130]. Women collegiate soccer increased from 1855 athletes on 80 teams during the 1981–1982 seasons to 22,682 athletes on 956 teams during the 2007–2008 seasons, making women’s soccer the NCAA sport with the greatest number of athletes [131]. A study of 2016 [132] showed that elite female soccer athletes are susceptible to stress fractures and menstrual dysfunction and experience delayed onset of menarche despite normal BMI and appropriate body perception and attitudes toward eating. Education about the detrimental effects of menstrual dysfunction and the importance of adequate energy balance and nutritional requirements should be encouraged to minimize the risk for poor bone health, manifesting as a stress fracture in the short term and osteoporosis over the long term in these athletes [132, 133].

The typical history of a stress fracture is localized pain of insidious onset which is initially not present at the start but occurs toward the end of a run. A sign of a more advanced fracture is pain progressing to occur during non-running-related activities, affecting day-to-day walking [126].

Defining the causative risk factors for stress fractures is difficult because there are many interrelated variables which make risk assessment problematic to study independently. Extrinsic and intrinsic factors may lead to stress fractures [126, 133, 134]. An increase in frequency, duration, or intensity of training load is often cited as a primary risk factor [135]. Hard training surfaces are also factors associated with lower-limb overuse injuries [135]. Training in shoes older than 6 months is a risk factor for stress fractures, likely related to the decrement in shock absorption as shoes age [135].

Regarding the intrinsic factors, Bennell et al. [22] demonstrated that smaller calf girth and less muscle mass in the lower limb of female runners was associated with a higher incidence of stress fractures. Kinematic and kinetic biomechanical variables have also been recently studied as potential risk factors for stress fractures; for example, in runners, an excessive hip adduction and rear-foot eversion are predictors of tibial stress fractures [136].

The hallmark physical examination finding is focal bony tenderness. Overlying swelling, erythema, or warmth are other potential examination findings. Less sensitive tests for fractures of long bones include the fulcrum test and hop test [137]. A functional kinetic chain assessment is useful to elucidate biomechanical factors that may predispose the runner to injury. Evaluating muscle imbalances, leg-length discrepancies, foot mechanics, genu varum, and femoral anteversion is appropriate because all have been associated with stress fractures [137].

Radiographic imaging should be used to supplement the clinical history and physical examination if uncertainty persists. Imaging can also be used to grade the severity of an injury and can thus be helpful in guiding treatment. CT is best used to differentiate lesions seen on a bone scan that may mimic stress fracture, including osteoid osteoma, osteomyelitis, and malignancy [138]. MRI is becoming the imaging study of choice, with many considering MRI the gold standard for the evaluation of bony stress injuries [139].

It is not only important to understand the significance of protection and rest, but also to understand the predisposing factors to the injury. Treatment is the time to explore and treat the contributing risk factors. For example, if low bone density is found, appropriate treatment is mandatory; if biomechanical issues are identified, and inappropriate shoes and training are determined, and specific rehabilitation is required [126].

Therapeutic ultrasound and electrical stimulation are purported modalities for enhancing the healing rate of fractures. Therapeutic ultrasound has been demonstrated to decrease healing time in acute tibial shaft, in distal radius fractures, and in navicular stress injuries [140, 141]. Electrical stimulation for bone growth has some support in delayed unions and non-unions, but only in uncontrolled trials for stress fractures [142].

### Juvenile osteochondritis dissecans (JOCD)

JOCD is a frequent cause of knee pain in adolescent athletes and non-athletes, with an incidence higher in boys than in girls [143, 144] and with delamination and localized necrosis of the subchondral bone. The etiology remains unclear [28, 29, 31]. Repetitive microtrauma, such as that of overuse injury, is considered the significant factor leading to JOCD [28, 29, 145].

The most common site of JOCD is the medial femoral condyle, accounting for 85% of the cases [28]. The term “osteochondritis” suggests an inflammatory etiology: however, histology shows damage of the bone and cartilage with no inflammation [146]. Local bone vascular insufficiency is also postulated to contribute to JOCD [29].

Highly active athletes present with a history of aching and gradual onset of knee pain of several days to weeks duration, typically located over the anterior portion of the knee, worse during activity. There may be a history of intermittent knee effusion following a practice or game session [29].

The examination may reveal mild effusion or limitation of motion of the knee. Findings may also vary depending on the stage of the disease [147]. In the early stages, with the articular cartilage over the femoral condyle still intact, the signs are non-specific. In the later stages, when the articular cartilage is eroded, the fragment may separate and become an intra-articular loose body. This can cause pain, effusion, and locking. Typically, in lesions, the medial femoral condyle when the athlete flexes and internally rotates the leg, from full extension to about 30°, pain is elicited and is relieved upon external rotation [28, 29].

Radiographic examination, with comparison with the other knee, is indicated when JOCD is suspected. In addition to the anteroposterior (AP) and lateral views, a tunnel view is useful to better identify the lesion, which appears as a well-demarcated radiolucent area [29, 31]. In those who demonstrate significant edema, a hemarthrosis or discomfort, and inability to bear weight without pain, an MRI is often obtained. An MRI can be helpful in identifying unstable lesions [31].

Suzue et al. [148] investigated the prevalence of JOCD in children and adolescent soccer players using a questionnaire, distributed to 1162 players. Of these, 547 patients experienced pain in the legs or lumbar spine. Radiographic or ultrasonographic examination was performed in 106 players, and 80 (75.5%) were diagnosed JOCD. In conclusion, the majority of players who had experienced pain and were found to have osteochondritis had severe injuries such as JOCD or lumbar spondylolysis [148].

Early diagnosis followed by restriction of activities and symptomatic treatment of pain generally allows for healing of lesions over a period of 8–12 weeks [30, 31, 149]. Spontaneous healing of the lesion is the usual outcome in children and adolescents with open distal femoral physis. Prognosis is excellent in younger patients [149].

Treatment is based on the stability of the lesion and the status of the overlying cartilage. The lesion may be unstable or loose, and these cases as well as in those athletes with large effusions or with marked symptoms which do not improve with conservative care may go to surgery for drilling, reattachment, or excision of the osteochondral lesion [31].

## Conclusions

Overuse injuries can affect the muscle, tendon, and bone. Tendon injuries give rise to substantial morbidity, and current understanding of the mechanisms involved in tendon injury and repair is limited. Tendon physiology and structure may include ROS involvement in various aspects of the predisposition to and participation in the degenerative process and subsequent response to injury. Bone can be damaged by repeated microtrauma and overuse. Stress reaction and stress fractures are very common in athletes, and the treatment consists in the treatment of the risk factors. Further research is required to improve our knowledge of tendon and bone healing. This will enable specific treatment strategies to be developed.

## Abbreviations

AP: Anteroposterior
AT: Achilles tendinopathy
BMD: Bone mineral density
FDA: Food and Drug Administration
HVIGI: High-volume image-guided injections
IL: Interleukin
JOCD: Juvenile osteochondritis dissecans
LIPUS: Low-intensity pulsed ultrasound
MCP: Monocyte chemoattractant protein
PCD: Programed cell death
PEMFs: Pulsed electromagnetic fields
PRP: Platelet-rich plasma
RLH: Royal London Hospital
RNS: Reactive nitrogen species
ROS: Reactive oxygen species
SW: Shock wave
TNF: Tumor necrosis factor
US: Ultrasound

## References

[1] Chéron C, Le Scanff C, Leboeuf-Yde C. Association between sports type and overuse injuries of extremities in adults: a systematic review. Chiropr Man Ther. 2017;25 Available from: http://chiromt.biomedcentral.com/articles/10.1186/s12998-017-0135-1. [cited 2017 Nov 8]. 10.1186/s12998-017-0135-1 PMC5237127 28101329

[2] Ekstrand J, Hagglund M, Walden M (2011). Injury incidence and injury patterns in professional football: the UEFA injury study. Br J Sports Med.

[3] Windt J, Gabbett TJ (2017). How do training and competition workloads relate to injury? The workload—injury aetiology model. Br J Sports Med.

[4] Gabbett TJ, Kennelly S, Sheehan J, Hawkins R, Milsom J, King E (2016). If overuse injury is a ‘training load error’, should undertraining be viewed the same way?. Br J Sports Med.

[5] Khan KM, Maffulli N (1998). Tendinopathy: an Achilles’ heel for athletes and clinicians. Clin J Sport Med.

[6] Maffulli N, Khan KM, Puddu G (1998). Overuse tendon conditions: time to change a confusing terminology. Arthrosc J Arthrosc Relat Surg Off Publ Arthrosc Assoc N Am Int Arthrosc Assoc.

[7] Magra M, Maffulli N (2005). Matrix metalloproteases: a role in overuse tendinopathies. Br J Sports Med.

[8] Sharma P, Maffulli N (2006). Biology of tendon injury: healing, modeling and remodeling. J Musculoskelet Neuronal Interact.

[9] Abate M, Silbernagel KG, Siljeholm C, Di Iorio A, De Amicis D, Salini V (2009). Pathogenesis of tendinopathies: inflammation or degeneration?. Arthritis Res Ther.

[10] Rees JD, Maffulli N, Cook J (2009). Management of tendinopathy. Am J Sports Med.

[11] Longo G, Ripalda P, Denaro V, Forriol F (2006). Morphologic comparison of cervical, thoracic, lumbar intervertebral discs of cynomolgus monkey (Macaca fascicularis). Eur Spine J.

[12] Gigante A, Specchia N, Rapali S, Ventura A, de Palma L (1996). Fibrillogenesis in tendon healing: an experimental study. Boll Della Soc Ital Biol Sper.

[13] Sharma P, Maffulli N (2005). Basic biology of tendon injury and healing. Surgeon.

[14] Banes AJ, Tsuzaki M, Yamamoto J, Fischer T, Brigman B, Brown T (1995). Mechanoreception at the cellular level: the detection, interpretation, and diversity of responses to mechanical signals. Biochem Cell Biol Biochim Biol Cell.

[15] Longo UG, Ronga M, Maffulli N (2009). Achilles tendinopathy. Sports Med Arthrosc Rev.

[16] Longo UG, Franceschi F, Ruzzini L, Rabitti C, Morini S, Maffulli N (2007). Light microscopic histology of supraspinatus tendon ruptures. Knee Surg Sports Traumatol Arthrosc.

[17] Maffulli N, Kenward MG, Testa V, Capasso G, Regine R, King JB (2003). Clinical diagnosis of Achilles tendinopathy with tendinosis. Clin J Sport Med Off J Can Acad Sport Med.

[18] Kvist M, Józsa L, Järvinen M, Kvist H (1985). Fine structural alterations in chronic Achilles paratenonitis in athletes. Pathol Res Pract.

[19] Aicale Rocco, Tarantino Domiziano, Maffulli Nicola (2017). Basic Science of Tendons. Bio-orthopaedics.

[20] Ackerman KE, Nazem T, Chapko D, Russell M, Mendes N, Taylor AP (2011). Bone microarchitecture is impaired in adolescent amenorrheic athletes compared with eumenorrheic athletes and nonathletic controls. J Clin Endocrinol Metab.

[21] Ackerman KE, Sokoloff NC, De NM, Clarke HM, Lee H, Misra M (2015). Fractures in relation to menstrual status and bone parameters in young athletes. Med Sci Sports Exerc.

[22] Bennell KL, Malcolm SA, Thomas SA, Wark JD, Brukner PD (1996). The incidence and distribution of stress fractures in competitive track and field athletes: a twelve-month prospective study. Am J Sports Med.

[23] Difiori JP, Benjamin HJ, Brenner JS, Gregory A, Jayanthi N, Landry GL (2014). Overuse injuries and burnout in youth sports: a position statement from the American Medical Society for Sports Medicine. Br J Sports Med.

[24] Tenforde AS, Sayres LC, McCurdy ML, Collado H, Sainani KL, Fredericson M (2011). Overuse injuries in high school runners: lifetime prevalence and prevention strategies. PM R.

[25] Changstrom BG, Brou L, Khodaee M, Braund C, Comstock RD (2015). Epidemiology of stress fracture injuries among us high school athletes, 2005–2006 through 2012–2013. Am J Sports Med.

[26] Goolsby MA, Barrack MT, Nattiv A (2012). A displaced femoral neck stress fracture in an amenorrheic adolescent female runner. Sports Health.

[27] Barrack MT, Gibbs JC, De S, Williams NI, Nichols JF, Rauh MJ (2014). Higher incidence of bone stress injuries with increasing female athlete triad-related risk factors: a prospective multisite study of exercising girls and women. Am J Sports Med.

[28] Peters TA, McLean LD (2000). Osteochondritis dissecans of the patellofemoral joint. Am J Sports Med.

[29] Pascual-Garrido C, Moran CJ, Green DW, Cole BJ (2013). Osteochondritis dissecans of the knee in children and adolescents. Curr Opin Pediatr.

[30] Schulz JF, Chambers HG (2013). Juvenile osteochondritis dissecans of the knee: current concepts in diagnosis and management. Instr Course Lect.

[31] Kocher MS, Tucker R, Ganley TJ, Flynn JM (2006). Management of osteochondritis dissecans of the knee: current concepts review. Am J Sports Med.

[32] Ramchurn N, Mashamba C, Leitch E, Arutchelvam V, Narayanan K, Weaver J (2009). Upper limb musculoskeletal abnormalities and poor metabolic control in diabetes. Eur J Intern Med.

[33] Hirai Shizuka, Takahashi Nobuyuki, Goto Tsuyoshi, Lin Shan, Uemura Taku, Yu Rina, Kawada Teruo (2010). Functional Food Targeting the Regulation of Obesity-Induced Inflammatory Responses and Pathologies. Mediators of Inflammation.

[34] Gaida JE, Ashe MC, Bass SL, Cook JL (2009). Is adiposity an under-recognized risk factor for tendinopathy? A systematic review. Arthritis Care Res.

[35] Wenner M (2009). Inflammatory clues. Sci Am.

[36] Almekinders LC, Temple JD (1998). Etiology, diagnosis, and treatment of tendonitis: an analysis of the literature. Med Sci Sports Exerc.

[37] Lewis JS, Sandford FM (2009). Rotator cuff tendinopathy: is there a role for polyunsaturated fatty acids and antioxidants?. J Hand Ther.

[38] Karousou E, Ronga M, Vigetti D, Barcolli D, Passi A, Maffulli N (2010). Molecular interactions in extracellular matrix of tendon. Front Biosci - Elite.

[39] Mavrikakis ME, Drimis S, Kontoyannis DA, Rasidakis A, Moulopoulou ES, Kontoyannis S (1989). Calcific shoulder periarthritis (tendinitis) in adult onset diabetes mellitus: a controlled study. Ann Rheum Dis.

[40] Longo UG, Olivia F, Denaro V, Maffulli N (2008). Oxygen species and overuse tendinopathy in athletes. Disabil Rehabil.

[41] Nespolo M. Free radicals in biology and medicine. Fifth Edition. by Barry Halliwell and John M. C. Gutteridge. Oxford University Press, 2015. Pp. xxxviii + 905. Price GBP 70.00 (paperback, ISBN 9780198717485), GBP 125.00 (hardback, ISBN 9780198717478). Acta Crystallogr Sect Struct Biol. 2017;73:384–5.

[42] Paoloni JA, Appleyard RC, Nelson J, Murrell GAC (2005). Topical glyceryl trinitrate application in the treatment of chronic supraspinatus tendinopathy: a randomized, double-blinded, placebo-controlled clinical trial. Am J Sports Med.

[43] Harper M-E, Bevilacqua L, Hagopian K, Weindruch R, Ramsey JJ (2004). Ageing, oxidative stress, and mitochondrial uncoupling. Acta Physiol Scand.

[44] Szomor ZL, Appleyard RC, Murrell GAC (2006). Overexpression of nitric oxide synthases in tendon overuse. J Orthop Res.

[45] Flowers F, Zimmerman JJ (1998). Reactive oxygen species in the cellular pathophysiology of shock. New Horiz Sci Pract Acute Med.

[46] Dennery PA (2006). Introduction to serial review on the role of oxidative stress in diabetes mellitus. Free Radic Biol Med.

[47] Burdon RH, Gill V, Rice-Evans C (1989). Cell proliferation and oxidative stress. Free Radic Res.

[48] Chénais B, Andriollo M, Guiraud P, Belhoussine R, Jeannesson P (2000). Oxidative stress involvement in chemically induced differentiation of K562 cells. Free Radic Biol Med.

[49] Clanton TL, Zuo L, Klawitter P (1999). Oxidants and skeletal muscle function: physiologic and pathophysiologic implications. Proc Soc Exp Biol Med.

[50] Gabbita SP, Robinson KA, Stewart CA, Floyd RA, Hensley K (2000). Redox regulatory mechanisms of cellular signal transduction. Arch Biochem Biophys.

[51] Jabs T (1999). Reactive oxygen intermediates as mediators of programmed cell death in plants and animals. Biochem Pharmacol.

[52] Lander HM (1997). An essential role for free radicals and derived species in signal transduction. FASEB J.

[53] Szabó C (1996). DNA strand breakage and activation of poly-ADP ribosyltransferase: a cytotoxic pathway triggered by peroxynitrite. Free Radic Biol Med.

[54] Göktürk E, Turgut A, Baygu C, Gunal I, Seber S, Gulbas Z (1995). Oxygen-free radicals impair fracture healing in rats. Acta Orthop.

[55] Halliwell B (1996). Oxidative stress, nutrition and health. Experimental strategies for optimization of nutritional antioxidant intake in humans. Free Radic Res.

[56] Ji LL (1999). Antioxidants and oxidative stress in exercise. Proc Soc Exp Biol Med.

[57] Aruoma OI (1994). Free radicals and antioxidant strategies in sports. J Nutr Biochem.

[58] Ji LL, Leeuwenburgh C, Leichtweis S, Gore M, Fiebig R, Hollander J (1998). Oxidative stress and aging. Role of exercise and its influences on antioxidant systems. Ann N Y Acad Sci.

[59] Hack V, Strobel G, Rau J-P, Weicker H (1992). The effect of maximal exercise on the activity of neutrophil granulocytes in highly trained athletes in a moderate training period. Eur J Appl Physiol.

[60] Goodship AE, Birch HL, Wilson AM (1994). The pathobiology and repair of tendon and ligament injury. Vet Clin North Am Equine Pract.

[61] Poli G, Parola M (1996). Oxidative damage and fibrogenesis. Free Radic Biol Med.

[62] Murrell GAC (2007). Using nitric oxide to treat tendinopathy. Br J Sports Med.

[63] Wang T-S, Kuo C-F, Jan K-Y, Huang H (1996). Arsenite induces apoptosis in Chinese hamster ovary cells by generation of reactive oxygen species. J Cell Physiol.

[64] Briehl MM, Baker AF (1996). Modulation of the antioxidant defence as a factor in apoptosis. Cell Death Differ.

[65] Morel Y, Barouki R (1999). Repression of gene expression by oxidative stress. Biochem J.

[66] Maffulli N, Longo UG, Loppini M, Denaro V (2010). Current treatment options for tendinopathy. Expert Opin Pharmacother.

[67] Maffulli N, Longo UG, Denaro V (2010). Novel approaches for the management of tendinopathy. J Bone Jt Surg - Ser A.

[68] Järvinen M, Józsa L, Kannus P, Järvinen TLN, Kvist M, Leadbetter W (1997). Histopathological findings in chronic tendon disorders. Scand J Med Sci Sports.

[69] Waterston SW (1997). Subcutaneous rupture of the Achilles tendon: basic science and some aspects of clinical practice. Br J Sports Med.

[70] Maffulli N (2007). Re: etiologic factors associated with symptomatic Achilles tendinopathy. Foot Ankle Int Am Orthop Foot Ankle Soc Swiss Foot Ankle Soc.

[71] Maffulli N, Kader D (2002). Tendinopathy of tendo Achillis. J Bone Jt Surg - Ser B.

[72] Clancy WG, Neidhart D, Brand RL (1976). Achilles tendonitis in runners: a report of five cases. Am J Sports Med.

[73] Williams JG (1993). Achilles tendon lesions in sport. Sports Med Auckl NZ.

[74] Denstad TF, Roaas A (1979). Surgical treatment of partial Achilles tendon rupture. Am J Sports Med.

[75] Fox JM, Blazina ME, Jobe FW, Kerlan RK, Carter VS, Shields J (1975). Degeneration and rupture of the Achilles tendon. CLINORTHOP.

[76] Magra M, Maffulli N (2008). Genetic aspects of tendinopathy. J Sci Med Sport.

[77] Sharma P, Maffulli N (2005). Tendon injury and tendinopathy: healing and repair. J Bone Joint Surg Am.

[78] Waldecker U, Hofmann G, Drewitz S (2012). Epidemiologic investigation of 1394 feet: coincidence of hindfoot malalignment and Achilles tendon disorders. Foot Ankle Surg Off J Eur Soc Foot Ankle Surg.

[79] Albers IS, Zwerver J, Diercks RL, Dekker JH, Van den Akker-Scheek I (2016). Incidence and prevalence of lower extremity tendinopathy in a Dutch general practice population: a cross sectional study. BMC Musculoskelet Disord.

[80] Renstrom P (1991). Sports traumatology today: a review of common current sports injury problems. Ann Chir Gynaecol.

[81] Kujala UM, Kvist M, Österman K (1986). Knee injuries in athletes: review of exertion injuries and retrospective study of outpatient sports clinic material. Sports Med.

[82] Astrom M, Rausing A. Chronic Achilles tendinopathy: a survey of surgical and histopathologic findings. Clin Orthop. 1995;316:151–64. 7634699

[83] Chard MD, Cawston TE, Riley GP, Gresham GA, Hazleman BL (1994). Rotator cuff degeneration and lateral epicondylitis: a comparative histological study. Ann Rheum Dis.

[84] Khan KM, Cook JL, Bonar F, Harcourt P, Åstrom M (1999). Histopathology of common tendinopathies: update and implications for clinical management. Sports Med.

[85] Longo UG, Rittweger J, Garau G, Radonic B, Gutwasser C, Gilliver SF (2009). No influence of age, gender, weight, height, and impact profile in Achilles tendinopathy in masters track and field athletes. Am J Sports Med.

[86] Järvinen TAH, Kannus P, Maffulli N, Khan KM (2005). Achilles tendon disorders: etiology and epidemiology. Foot Ankle Clin.

[87] Fredberg U, Bolvig L, Andersen NT (2008). Prophylactic training in asymptomatic soccer players with ultrasonographic abnormalities in Achilles and patellar tendons: the Danish super league study. Am J Sports Med.

[88] Alfredson H, Ohberg L (2005). Neovascularisation in chronic painful patellar tendinosis--promising results after sclerosing neovessels outside the tendon challenge the need for surgery. Knee Surg Sports Traumatol Arthrosc Off J ESSKA..

[89] Woods C, Hawkins R, Hulse M, Hodson A (2002). The Football Association Medical Research Programme: an audit of injuries in professional football-analysis of preseason injuries. Br J Sports Med.

[90] Sobhani S, Dekker R, Postema K, Dijkstra PU (2013). Epidemiology of ankle and foot overuse injuries in sports: a systematic review. Scand J Med Sci Sports.

[91] Leadbetter WB (1992). Cell-matrix response in tendon injury. Clin Sports Med.

[92] Ilfeld FW. Can stroke modification relieve tennis elbow? Clin Orthop. 1992;276:182–6. 1537149

[93] Kibler WB, Chandler TJ, Pace BK (1992). Principles of rehabilitation after chronic tendon injuries. Clin Sports Med.

[94] MG JF and C. Lateral and medial epicondylitis of the elbow. - PubMed - NCBI [Internet]. Available from: https://www.ncbi.nlm.nih.gov/pubmed/10708988. [cited 2017 Nov 3]

[95] Almekinders LC, Gilbert JA (1986). Healing of experimental muscle strains and the effects of nonsteroidal antiinflammatory medication. Am J Sports Med.

[96] Garrett WE (1990). Muscle strain injuries: clinical and basic aspects. Med Sci Sports Exerc.

[97] Dean BJF, Franklin SL, Carr AJ (2013). The peripheral neuronal phenotype is important in the pathogenesis of painful human tendinopathy: a systematic review. Clin Orthop..

[98] Hutchison A-M, Evans R, Bodger O, Pallister I, Topliss C, Williams P (2013). What is the best clinical test for Achilles tendinopathy?. Foot Ankle Surg Off J Eur Soc Foot Ankle Surg..

[99] Maffulli N, Testa V, Capasso G, Oliva F, Sullo A, Benazzo F (2006). Surgery for chronic Achilles tendinopathy yields worse results in nonathletic patients. Clin J Sport Med Off J Can Acad Sport Med..

[100] Maffulli N, Wong J, Almekinders LC (2003). Types and epidemiology of tendinopathy. Clin Sports Med.

[101] Ohberg L, Lorentzon R, Alfredson H (2001). Neovascularisation in Achilles tendons with painful tendinosis but not in normal tendons: an ultrasonographic investigation. Knee Surg Sports Traumatol Arthrosc Off J ESSKA..

[102] Rolf C, Movin T (1997). Etiology, histopathology, and outcome of surgery in achillodynia. Foot Ankle Int.

[103] Maffulli N, Oliva F, Maffulli GD, Giai Via A, Gougoulias N (2017). Minimally invasive Achilles tendon stripping for the management of tendinopathy of the main body of the Achilles tendon. J Foot Ankle Surg Off Publ Am Coll Foot Ankle Surg.

[104] Silbernagel KG, Brorsson A, Lundberg M (2011). The majority of patients with Achilles tendinopathy recover fully when treated with exercise alone: a 5-year follow-up. Am J Sports Med.

[105] Weinreb JH, Sheth C, Apostolakos J, McCarthy M-B, Barden B, Cote MP (2014). Tendon structure, disease, and imaging. Muscles Ligaments Tendons J.

[106] Maffulli N, Via AG, Oliva F (2015). Chronic Achilles tendon disorders. Tendinopathy and Chronic Rupture Clin Sports Med.

[107] Sayana MK, Maffulli N (2007). Eccentric calf muscle training in non-athletic patients with Achilles tendinopathy. J Sci Med Sport.

[108] Alfredson H, Pietilä T, Jonsson P, Lorentzon R (1998). Heavy-load eccentric calf muscle training for the treatment of chronic Achilles tendinosis. Am J Sports Med.

[109] Rompe JD, Nafe B, Furia JP, Maffulli N (2007). Eccentric loading, shock-wave treatment, or a wait-and-see policy for tendinopathy of the main body of tendo Achillis: a randomized controlled trial. Am J Sports Med.

[110] van Sterkenburg MN, van Dijk CN (2011). Injection treatment for chronic midportion Achilles tendinopathy: do we need that many alternatives?. Knee Surg Sports Traumatol Arthrosc Off J ESSKA..

[111] Maffulli N, Papalia R, D’Adamio S, Diaz Balzani L, Denaro V (2015). Pharmacological interventions for the treatment of Achilles tendinopathy: a systematic review of randomized controlled trials. Br Med Bull.

[112] van Sterkenburg MN, de Jonge MC, Sierevelt IN, van Dijk CN (2010). Less promising results with sclerosing ethoxysclerol injections for midportion Achilles tendinopathy: a retrospective study. Am J Sports Med.

[113] Ebbesen BH, Mølgaard CM, Olesen JL, Gregersen HE, Simonsen O (2018). No beneficial effect of polidocanol treatment in Achilles tendinopathy: a randomised controlled trial. Knee Surg Sports Traumatol Arthrosc Off J ESSKA..

[114] de Vos RJ, Weir A, van Schie HTM, Bierma-Zeinstra SMA, Verhaar JAN, Weinans H (2010). Platelet-rich plasma injection for chronic Achilles tendinopathy: a randomized controlled trial. JAMA.

[115] Chan O, O’Dowd D, Padhiar N, Morrissey D, King J, Jalan R (2008). High volume image guided injections in chronic Achilles tendinopathy. Disabil Rehabil.

[116] Abate Michele, Di Carlo Luigi, Verna Sandra, Di Gregorio Patrizia, Schiavone Cosima, Salini Vincenzo (2018). Synergistic activity of platelet rich plasma and high volume image guided injection for patellar tendinopathy. Knee Surgery, Sports Traumatology, Arthroscopy.

[117] Paavola M, Kannus P, Paakkala T, Pasanen M, Järvinen M (2000). Long-term prognosis of patients with achilles tendinopathy. An observational 8-year follow-up study. Am J Sports Med.

[118] Longo UG, Ramamurthy C, Denaro V, Maffulli N (2008). Minimally invasive stripping for chronic Achilles tendinopathy. Disabil Rehabil.

[119] Alfredson H (2011). Ultrasound and Doppler-guided mini-surgery to treat midportion Achilles tendinosis: results of a large material and a randomised study comparing two scraping techniques. Br J Sports Med.

[120] Testa V, Capasso G, Benazzo F, Maffulli N (2002). Management of Achilles tendinopathy by ultrasound-guided percutaneous tenotomy. Med Sci Sports Exerc.

[121] Maffulli N, Testa V, Capasso G, Bifulco G, Binfield PM (1997). Results of percutaneous longitudinal tenotomy for Achilles tendinopathy in middle- and long-distance runners. Am J Sports Med.

[122] Gajhede-Knudsen M, Ekstrand J, Magnusson H, Maffulli N (2013). Recurrence of Achilles tendon injuries in elite male football players is more common after early return to play: an 11-year follow-up of the UEFA Champions League injury study. Br J Sports Med.

[123] Schultz W, Stinus H, Schleicher W, Hess T (1991). Stress reactions--stress fracture of the upper femoral neck in endurance sports. Sportverletz Sportschaden Organ Ges Orthopadisch-Traumatol Sportmed.

[124] Armstrong DW, Rue J-PH, Wilckens JH, Frassica FJ (2004). Stress fracture injury in young military men and women. Bone.

[125] Fullem BW (2015). Overuse lower extremity injuries in sports. Clin Podiatr Med Surg.

[126] Harrast MA, Colonno D (2010). Stress fractures in runners. Clin Sports Med.

[127] Wright AA, Hegedus EJ, Lenchik L, Kuhn KJ, Santiago L, Smoliga JM (2016). Diagnostic accuracy of various imaging modalities for suspected lower extremity stress fractures: a systematic review with evidence-based recommendations for clinical practice. Am J Sports Med.

[128] Rizzone KH, Ackerman KE, Roos KG, Dompier TP, Kerr ZY. Epidemiology of stress fractures in collegiate student-athletes, 2004–2005 through 2013–2014 academic years. J Athl Train. 2017; Available from: http://natajournals.org/doi/10.4085/1062-6050-52.8.01. [cited 2017 Nov 5]. 10.4085/1062-6050-52.8.01 PMC5687241 28937802

[129] Giza E, Mithöfer K, Farrell L, Zarins B, Gill T (2005). Injuries in women’s professional soccer. Br J Sports Med.

[130] Associations NF of SHS. High School Athletics Participation Survey 2011–2012. National Federation of State High School Associations Indianapolis, IN; 2012.

[131] NCAA Publications - 1981-82 - 2008-09 NCAA Sports Sponsorship and Participation Rates Report [Internet]. [cited 2018 Dec 1]. Available from: https://www.ncaapublications.com/p-4177-1981-82-2008-09-ncaa-sports-sponsorship-and-participation-rates-report.aspx

[132] Prather H, Hunt D, McKeon K, Simpson S, Meyer EB, Yemm T (2016). Are elite female soccer athletes at risk for disordered eating attitudes, menstrual dysfunction, and stress fractures?. PM R.

[133] Wright AA, Taylor JB, Ford KR, Siska L, Smoliga JM (2015). Risk factors associated with lower extremity stress fractures in runners: a systematic review with meta-analysis. Br J Sports Med.

[134] Bowerman EA, Whatman C, Harris N, Bradshaw E (2015). A review of the risk factors for lower extremity overuse injuries in young elite female ballet dancers. J Dance Med Sci Off Publ Int Assoc Dance Med Sci.

[135] Johanson MA (1992). Contributing factors in microtrauma injuries of the lower extremity. J Back Musculoskelet Rehabil.

[136] Pohl MB, Mullineaux DR, Milner CE, Hamill J, Davis IS (2008). Biomechanical predictors of retrospective tibial stress fractures in runners. J Biomech.

[137] James SL, Bates BT, Osternig LR (1978). Injuries to runners. Am J Sports Med.

[138] Kiss ZS, Khan KM, Fuller PJ (1993). Stress fractures of the tarsal navicular bone: CT findings in 55 cases. Am J Roentgenol.

[139] Kiuru MJ, Pihlajamaki HK, Hietanen HJ, Ahovuo JA (2002). MR imaging, bone scintigraphy, and radiography in bone stress injuries of the pelvis and the lower extremity. Acta Radiol.

[140] Heckman JD, Ryaby JP, McCabe J, Frey JJ, Kilcoyne RF (1994). Acceleration of tibial fracture-healing by non-invasive, low-intensity pulsed ultrasound. J Bone Jt Surg - Ser A.

[141] Malliaropoulos N, Alaseirlis D, Konstantinidis G, Papalada A, Tsifountoudis I, Petras K (2017). Therapeutic ultrasound in navicular stress injuries in elite track and field athletes. Clin J Sport Med Off J Can Acad Sport Med..

[142] Rettig AC, Shelbourne KD, McCarroll JR, Bisesi M, Watts J (1988). The natural history and treatment of delayed union stress fractures of the anterior cortex of the tibia. Am J Sports Med.

[143] Kessler JI, Nikizad H, Shea KG, Jacobs JC, Bebchuk JD, Weiss JM (2014). The demographics and epidemiology of osteochondritis dissecans of the knee in children and adolescents. Am J Sports Med.

[144] Kumar V, Bhatnagar N, Lodhi JS (2018). Grade I osteochondritis dissecans in a young professional athlete. Indian J Orthop.

[145] Krause M, Hapfelmeier A, Möller M, Amling M, Bohndorf K, Meenen NM (2013). Healing predictors of stable juvenile osteochondritis dissecans knee lesions after 6 and 12 months of nonoperative treatment. Am J Sports Med.

[146] Rothermich MA, Glaviano NR, Li J, Hart JM (2015). Patellofemoral pain. Epidemiology, pathophysiology, and treatment options. Clin Sports Med.

[147] Uppstrom TJ, Gausden EB, Green DW (2016). Classification and assessment of juvenile osteochondritis dissecans knee lesions. Curr Opin Pediatr.

[148] Suzue N, Matsuura T, Iwame T, Hamada D, Goto T, Takata Y (2014). Prevalence of childhood and adolescent soccer-related overuse injuries. J Med Investig JMI.

[149] Yang JS, Bogunovic L, Wright RW (2014). Nonoperative treatment of osteochondritis dissecans of the knee. Clin Sports Med.

